# Supplementary material for: Effect of Template–Mediated Alumina Nanoparticle Morphology on Sapphire Wafer Production via Heat Exchange Method
Source: Materials (Basel). 2023 Aug 30;16(17):5938. doi: 10.3390/ma16175938 (PMC10488677; doi:10.3390/ma16175938)
Supplement: Supplementary file 1 [file materials-16-05938-s001.zip › materials-2389390-supplementary.pdf]

materials

Supporting information for

Effect of Template-Mediated Alumina Nanoparticle Morphology on Sapphire Wafer Production via Heat Exchange Method

Yadian Xie [1,*,†], Miaoxuan Xue [1,†], Lanxing Gao [1,†], Yanqing Hou [1,2], Bo Yang [1,2,3,*] and Xin Tong [1,3,*]

[1] Guizhou Provincial Key Laboratory in High Education Institutions of Low-Dimensional Materials and Environmental and Ecological Governance, Key Laboratory of Low-Dimensional Materials and Big Data, College of Chemical Engineering, Guizhou Minzu University, Guiyang 550025, China; xuemx0418@163.com (M.X.); glxprettylife@163.com (L.G.); hhouyanqing@163.com (Y.H.)
[2] Faculty of Metallurgy and Energy Engineering, Kunming University of Science and Technology, Kunming 650093, China
[3] School of Chemistry and Material Science, Guizhou Normal University, Guiyang 550014, China
* Correspondence: xieyadian@gzmu.edu.cn (Y.X.); tongxin@gznu.edu.cn (X.T.); gznuyangbo@163.com (B.Y.)
† These authors contributed equally to this work.

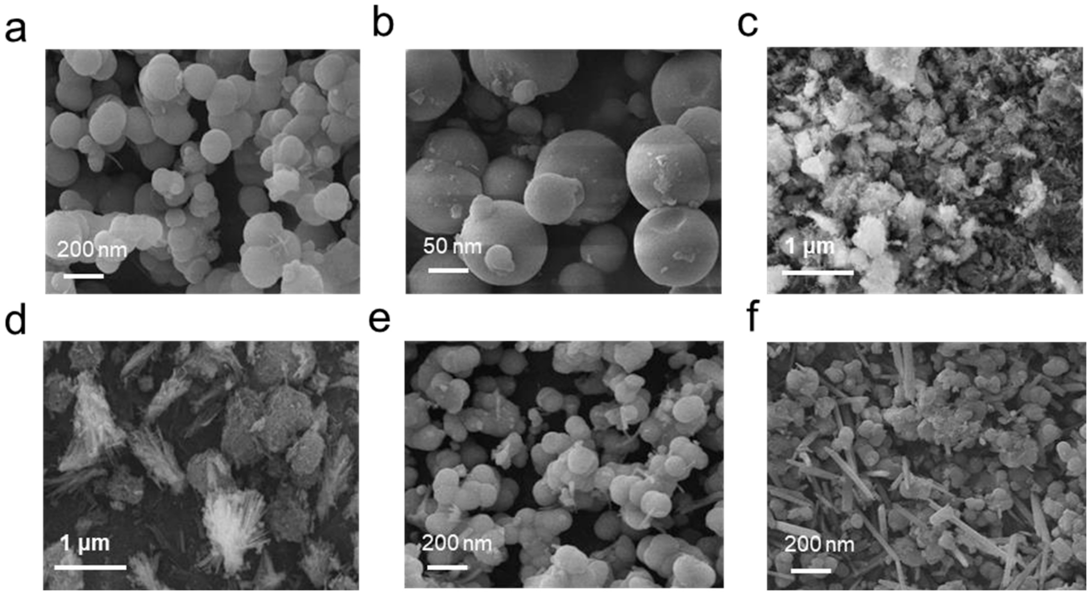

Figure S1. The intermediate Al_2O_3 SEM image under different synthesis conditions: a) Al_2O_3-100-3; b) Al_2O_3-130-3; c) Al_2O_3-160-3; d) Al_2O_3-190-3; e) Al_2O_3-130-6; f) Al_2O_3-130-9.

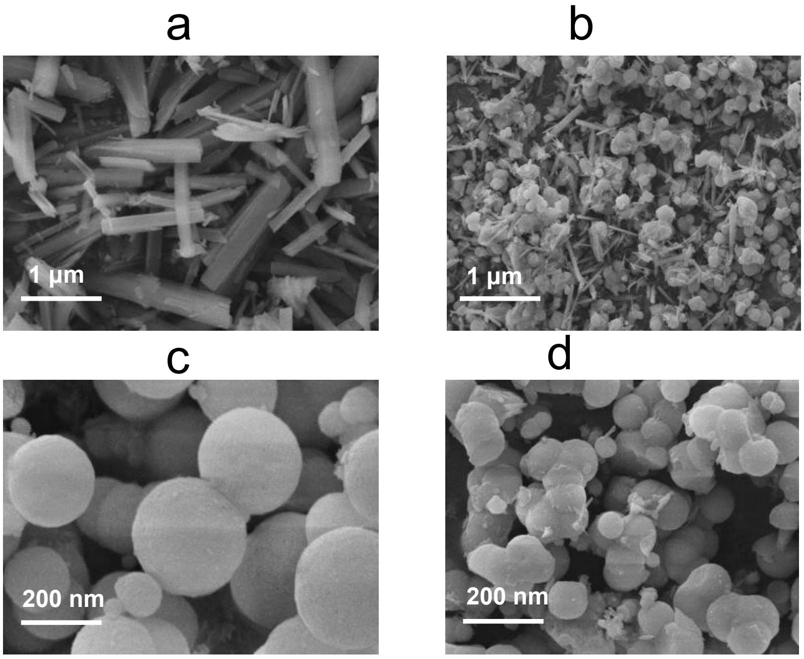

Figure S2. The intermediate Al_2O_3 SEM image prepared with different AA/urea weight ratios: a) Al_2O_3-0; b) Al_2O_3-0.625; c) Al_2O_3-1.25; d) Al_2O_3-2.5.
